# Combining Cre-LoxP and single-cell sequencing technologies: insights into the extracellular vesicle cargo transfer

**DOI:** 10.20517/evcna.2024.58

**Published:** 2024-11-09

**Authors:** Michael W. Pfaffl

**Affiliations:** Animal Physiology and Immunology, School of Life Sciences, Technical University of Munich, Freising 85354, Germany*.*

**Keywords:** Extracellular vesicles, intracellular cargo transport, pancreatic ductal adenocarcinoma, tumor microenvironments, Cre-LoxP, single-cell sequencing, Bag6

## Abstract

The recent study from the Pogge von Strandmann group published in *Cellular and Molecular Immunology*, by Alashkar Alhamwe *et al*., combined for the first time the Cre-LoxP recombination system with single-cell sequencing. The group monitored the tumor-derived extracellular vesicle (EV) uptake and the EV functions in the recipient non-malignant cells in a pancreatic ductal adenocarcinoma mouse model. Recombination events and EV uptake, together with resulting gene expression changes in macrophages, neutrophils, and mast cells, were detected by single-cell sequencing technology of the tumor tissue. This new approach is highly specific, as it can identify single EV recipient cells without interfering with the EV biogenesis or the phenotype.

## TEXT

Extracellular vesicles (EVs) are diverse, nanosized, double membrane-bound structures secreted by nearly all cell types under healthy and pathological conditions. They play a crucial role in mediating intercellular communication by transferring and delivering biologically active molecules, such as various types of nucleic acids, proteins, metabolites, and lipids, to adjacent tissues or distant recipient cells, thereby eliciting specific biological responses^[1]^.

Despite significant advancements in EV research during the last two decades, a major challenge impeding their diagnostic and therapeutic application is the limited understanding of their *in vivo* biological functions. Determining the EV biodistribution in tissues and organs and the underlying EV kinetics has been the primary focus of most *in vivo* studies, with a possible emphasis on their role in immune recipient cells. Based on the minimal information for studies of extracellular vesicles (MISEV) 2023 guideline, fluorescence and bioluminescent tags have been used to monitor tumors and EVs *in vivo* by labeling and tracing cells^[1-4]^. However, several disadvantages arise, including issues with EV labeling efficiency, deep tissue penetration, and signal-to-noise ratio. Furthermore, the type of labeling tag itself, e.g., using green fluorescent protein (GFP), can alter the EV biogenesis, the phenotype, and finally, the function. In addition, the use of additional genetic manipulation approaches, such as incorporating a tag fusion construct (e.g., CD63-GFP), may lead to increased off-target effects in non-malignant immune cells.

Today, the Cre-LoxP recombination system is widely established as a highly effective method to study EV uptake and function *in vivo*^[5-7]^. To visualize the uptake of EVs by recipient cells, a reporter mouse strain such as B6.129(Cg)-Gt(ROSA)26Sortm4(ACTB-tdTomato,-eGFP)Luo/J is used. If vesicles equipped with the Cre recombinase are taken up by target cells, the excision of a LoxP-flanked stop cassette upstream of eGFP occurs and enables eGFP expression. Green fluorescent target cells can then be identified, e.g., via immunohistochemistry of tissues or upon isolation using flow cytometry^[5-7]^. One recent tool to study the *in vivo* transfer of EVs is the Exomap1 transgenic mouse, which, in response to Cre recombinase, expresses an exosomal marker protein^[8]^. Using *in vivo* tumor models offers a deeper understanding of the direct transfer of EV cargo between malignant cells and, furthermore, from malignant to non-malignant cells within the tumor microenvironment (TME). However, so far, the identification and characterization of the fluorescent target cells remain technically challenging.

A recent study from the Pogge von Strandmann lab combined, for the first time, the Cre-LoxP system with a state-of-the-art single-cell sequencing approach to tackle this limitation. The aim of the study was to investigate the role of the immune cell regulator and chaperone BAG6 (Bcl2-associated-athanogene 6) in EV cargo loading and function within the pancreatic cancer TME^[9]^. To this end, the authors used a preclinical mouse model for pancreatic ductal adenocarcinoma (PDAC) in a BAG6 pro- or deficient background employing Cre-LoxP reporter mice. The uptake of Cre recombinase mRNA, delivered through EVs from transplanted tumor cells, was tracked at the molecular level by single-cell RNA sequencing (scRNAseq) of the tumor tissue. The beauty of the system is that the functional impact of EV uptake on the target cells can be directly analyzed at the single-cell level. This is possible by comparing the gene expression pattern of cells with and without vesicle uptake, which is indicated by recombination and, therefore, eGFP expression^[9]^. Combining the Cre-LoxP system with scRNA sequencing thus allows for determining the gene profile of target cells upon EV uptake in a direct comparison with non-recombined cells in the tissue. Thus, specific EV target cell populations can be identified, as the proteins they present offer a reliable tool for investigating the functional role of EVs and their impact on cellular signaling and downstream effects on various biological processes. The validity of the method was verified through immunofluorescence staining of GFP protein in the tumor tissues of animals transplanted with Cre+ or Cre- tumor cells.

Using this approach, the authors elegantly showed that the *in vivo* EV uptake induced changes in the cellular composition of the TME in a Bag6-dependent manner. Recombination and vesicle uptake were detectable, e.g., in macrophages and neutrophils, but of note, predominantly and specifically in the mast cell compartment of Bag6-deficient tumors. Further analysis revealed that this activation was triggered by the interaction between the Interleukin-33 receptor and Inteleukin-33 presenting tumor-derived EVs. This EV-initiated cascade induced changes within the TME, generating a tumor-promoting milieu and accelerated tumor growth. *In vivo* tracking of EVs involves labeling EVs or tumor cells with fluorescent dyes or incorporating tag fusion constructs, which can affect the biogenesis, integrity, surface properties, and biological activity of EVs. To address these limitations, the Cre-LoxP-based technique offers a precise method for monitoring EV transfer in both *in vitro* and *in vivo* models via fluorescent labeling to specifically determine EV target cells^[4]^. By using this approach together with single-cell sequencing, it is possible to track EV uptake *in vivo* and, at the same time, to investigate the impact of vesicle uptake on gene expression in the recipient cell. The authors applied the Pan02 model for their studies, which does not harbor the pancreatic cancer driver mutations Kirsten rat sarcoma viral oncogene homolog (KRAS), tumor protein 53 (TP53), and cyclin-dependent kinase inhibitor 2A (CDKN2A), but a SMAD family member 4 (SMAD4) mutation^[10,11]^. Pan02 cells thus express wild-type p53, which is known to be involved in Bag6-dependent EV biogenesis^[12]^. Therefore, it would be interesting to compare the EV phenotype of Pan02 tumors with other models with different genetic backgrounds with a view on PDAC heterogeneity. This method is not limited to PDAC mouse models but can be applied generally in research addressing the function of EVs in physiological conditions and diseases. The approach is highly specific, may identify single EV recipient cells, and unravels how tumor-derived EVs alter the phenotype of non-malignant immune cells in the TME. In the future, further developments are needed to elucidate the influence of proteins presented on the corona of the EVs, such as Interleukin-33, to elucidate the phenotype of recipient cells in the context of uptake-independent ligand-receptor interactions.

## DECLARATIONS

### Copyright

© The Author(s) 2024.
